# Supplementary material for: Microsecond interaural time difference discrimination restored by cochlear implants after neonatal deafness
Source: eLife. 2021 Jan 11;10:e59300. doi: 10.7554/eLife.59300 (PMC7815311; doi:10.7554/eLife.59300)
Supplement: Transparent reporting form [file elife-59300-transrepform.pdf]

eLIFE
1st Floor
24 Hills Road
Cambridge CB2 1JP, UK
P 01223 855340
W elifesciences.org
T @elife

eLife's transparent reporting form

We encourage authors to provide detailed information *within their submission* to facilitate the interpretation and replication of experiments. Authors can upload supporting documentation to indicate the use of appropriate reporting guidelines for health-related research (see EQUATOR Network), life science research (see the BioSharing Information Resource), or the ARRIVE guidelines for reporting work involving animal research. Where applicable, authors should refer to any relevant reporting standards documents in this form.

If you have any questions, please consult our Journal Policies and/or contact us: editorial@elifesciences.org.

Sample-size estimation

- You should state whether an appropriate sample size was computed when the study was being designed
- You should state the statistical method of sample size computation and any required assumptions
- If no explicit power analysis was used, you should describe how you decided what sample (replicate) size (number) to use

Please outline where this information can be found within the submission (e.g., sections or figure legends), or explain why this information doesn't apply to your submission:

This study is a pioneering, exploratory study investigating whether neonatally deaf rats would be able to become sensitive to ITDs, and what psychoacoustic thresholds they might be able to achieve. Since there are no other published studies that have ever shown behavioral ITD sensitivity to CI stimulation in an animal model, there was no basis for appropriate *a-priori* choices of likely "effect sizes". Furthermore, this is not a cohort study in which we were hoping to determine whether there were statistically significant differences between cohorts based on treatments. Rather, we were interested in determining, in the first instance, whether neonatally deaf rats can develop sensitivity to ITDs delivered over CIs at all. For these experiments, each animal is its own internal control, generating thousands of trials in which responses to binaural CI stimuli with left-leading vs. right-leading stimuli are compared. The statistical power of our work thus comes from the thousands of trials performed by each animal, and not from the sample size in the sense of the number of animals tested. Our figure 1 does offer a visual comparison of deaf and normally hearing animals to put our findings into context, but the objective of our study was not measure which of these cohorts does "better". Power calculations are therefore not appropriate for this type of study. Our choice to train and test 5 CI animals in this pioneering study was simply a compromise, small enough to be manageable given the considerable technical difficulty involved in implanting, training and testing each individual, but large enough to give a clear indication as to whether the results obtained are reproducible, as they indeed turned out to be given that all 5 animals implanted, trained and tested learned to lateralize sub-millisecond ITDs with accuracies that are simply astronomically unlikely to have arisen by chance.

eLife Sciences Publications, Ltd is a limited liability non-profit non-stock corporation incorporated in the State of Delaware, USA, with company number 5030732, and is registered in the UK with company number FC030576 and branch number BR015634 at the address 1st Floor, 24 Hills Road, Cambridge CB2 1JP | August 2014

eLIFE
1st Floor
24 Hills Road
Cambridge CB2 1JP, UK
P 01223 855340
W elifesciences.org
T @elife

Replicates

- You should report how often each experiment was performed
- You should include a definition of biological versus technical replication
- The data obtained should be provided and sufficient information should be provided to indicate the number of independent biological and/or technical replicates
- If you encountered any outliers, you should describe how these were handled
- Criteria for exclusion/inclusion of data should be clearly stated
- High-throughput sequence data should be uploaded before submission, with a private link for reviewers provided (these are available from both GEO and ArrayExpress)

Please outline where this information can be found within the submission (e.g., sections or figure legends), or explain why this information doesn't apply to your submission:

Our Materials & Methods and our Results sections contain detailed information answering all these questions about replication. In figure 1, our key data figure, we even have little numerical insets showing the reader at a glance how many individual trials went into every single data point. In our study cochlear implanted ND rats were trained at least 5 times a week twice a day and generated between 150-200 trials per session (see Materials & Methods, line 459-460). In total, the behavioral data were generated over a period of minimum at least 4 weeks. Each ND rat completed at least 40 test sessions with about 6000-8000 ITD lateralization trials. All animals were included in the study.

eLife Sciences Publications, Ltd is a limited liability non-profit non-stock corporation incorporated in the State of Delaware, USA, with company number 5030732, and is registered in the UK with company number FC030576 and branch number BR015634 at the address 1st Floor, 24 Hills Road, Cambridge CB2 1JP | August 2014

Statistical reporting

- Statistical analysis methods should be described and justified
- Raw data should be presented in figures whenever informative to do so (typically when N per group is less than 10)
- For each experiment, you should identify the statistical tests used, exact values of N, definitions of center, methods of multiple test correction, and dispersion and precision measures (e.g., mean, median, SD, SEM, confidence intervals; and, for the major substantive results, a measure of effect size (e.g., Pearson's r, Cohen's d)
- Report exact p-values wherever possible alongside the summary statistics and 95% confidence intervals. These should be reported for all key questions and not only when the p-value is less than 0.05.

Please outline where this information can be found within the submission (e.g., sections or figure legends), or explain why this information doesn't apply to your submission:
(For large datasets, or papers with a very large number of statistical tests, you may upload a single table file with tests, Ns, etc., with reference to sections in the manuscript.)

The statistical analysis of our behavioral data is described in detail in the section "Psychometric curve fitting" of the Method section (lines 548-591) and the results of the analysis are presented in figure 1 and explained in the Result section (lines 101-131). The analysis of the electrophysiological data is also described in the methods "Data anaylsis" (lines 518-536) and "Signal-to-noise ratio (SNR) calculation (lines 537-547) and presented in figures 2 and 3. The exact results of the analysis can be found in the Result section (lines 154-176; 191-219).

Group allocation

- Indicate how samples were allocated into experimental groups (in the case of clinical studies, please specify allocation to treatment method); if randomization was used, please also state if restricted randomization was applied
- Indicate if masking was used during group allocation, data collection and/or data analysis

Please outline where this information can be found within the submission (e.g., sections or figure legends), or explain why this information doesn't apply to your submission:

The collection and the analysis of both the behavioral and electrophysiological data was performed in a highly automated manner, using standardized python and MATLAB programs which are identical for the normally hearing and neonatally deafened animals, thus obviating the possibility of observer bias.

Before starting the experiments, the animals were randomly allocated to one of four experimental groups (lines 386-388). Nine rats were neonatally deafened (ND), and, on reaching young adulthood, 5 of these were trained for behavior, and the remaining 4 were used for acute electrophysiology. A further 9 rats, were allowed to mature with normal hearing experience, 5 of which were used in psychoacoustic training and another 4 were used for acute electrophysiology (Results section, lines 103-107 and 158-161).

Additional data files ("source data")

- We encourage you to upload relevant additional data files, such as numerical data that are represented as a graph in a figure, or as a summary table
- Where provided, these should be in the most useful format, and they can be uploaded as "Source data" files linked to a main figure or table
- Include model definition files including the full list of parameters used
- Include code used for data analysis (e.g., R, MatLab)
- Avoid stating that data files are "available upon request"

Please indicate the figures or tables for which source data files have been provided:

We will upload all the source data and analysis scripts to a public depository such as datadryad as soon as our paper is accepted for publication.

eLife Sciences Publications, Ltd is a limited liability non-profit non-stock corporation incorporated in the State of Delaware, USA, with company number 5030732, and is registered in the UK with company number FC030576 and branch number BR015634 at the address 1st Floor, 24 Hills Road, Cambridge CB2 1JP | August 2014
